# Case Report: Initial Treatment Adjustments and Complications in Ovarian Cancer Patient With Inborn Error of Immunity

**DOI:** 10.3389/fonc.2022.843741

**Published:** 2022-06-29

**Authors:** Jamila Mammadova, Anna Redden, Rachel Cruz, Boglarka Ujhazi, Sumai Gordon, Maryssa Ellison, Tyra Gatewood, Carla Duff, Anthony Cannella, Charurut Somboonwit, Chakrapol Sriaroon, Krisztian Csomos, Joseph F. Dasso, Terry Harville, Roohi Ismail-Khan, Jolan E. Walter

**Affiliations:** ^1^ Morsani College of Medicine, University of South Florida, Tampa, FL, United States; ^2^ Charles E. Schmidt College of Medicine, Florida Atlantic University, Boca Raton, FL, United States; ^3^ Division of Allergy and Immunology, Department of Pediatrics, Morsani College of Medicine, University of South Florida, Tampa, FL, United States; ^4^ Department of Pharmacy at Gynecologic and Neuro Oncology Clinics, H. Lee Moffitt Cancer Center and Research Institute, Tampa, FL, United States; ^5^ Division of Infectious Disease and International Medicine, Department of Internal Medicine, Morsani College of Medicine, University of South Florida, Tampa, FL, United States; ^6^ Division of Hematology, Department of Pathology and Laboratory Services, and Department of Internal Medicine, University of Arkansas for Medical Sciences, Little Rock, AR, United States; ^7^ Department of Cardio-Oncology, H. Lee Moffitt Cancer Center and Research Institute, Tampa, FL, United States

**Keywords:** antibody deficiency, primary immunodeficiency, ovarian cancer, case report, inborn error of immunity

## Abstract

**Background:**

Patients with inborn errors of immunity (IEI) have increased risk of developing cancers secondary to impaired anti-tumor immunity. Treatment of patients with IEI and cancer is challenging as chemotherapy can exacerbate infectious susceptibility. However, the literature on optimal cancer treatment in the setting of IEI is sparse.

**Objectives:**

We present a patient with specific antibody deficiency with normal immunoglobins (SADNI), immune dysregulation (ID), and stage III ovarian carcinoma as an example of the need to modify conventional treatment in the context of malignancy, IEI, and ongoing infections.

**Methods:**

This is a retrospective chart review of the patient’s clinical manifestations, laboratory evaluation and treatment course.

**Results:**

Our patient is a female with SADNI and ID diagnosed with stage III ovarian carcinoma at 60 years of age. Her ID accounted for antinuclear antibody positive (ANA+) mixed connective tissue diseases, polyarthralgia, autoimmune neutropenia, asthma, autoimmune thyroiditis, and Celiac disease. Due to the lack of precedent in the literature, her treatment was modified with continuous input from infectious disease, allergy/immunology and oncology specialist using a multidisciplinary approach.

The patient completed debulking surgery and 6 cycles of chemotherapy. The dosing for immunoglobulin replacement therapy was increased for prophylaxis. Chemotherapy doses were lowered for all cycles preemptively for IEI. The therapy included carboplatin, paclitaxel, bevacizumab, and pegfilgrastim. The patient completed six-months of maintenance medication involving bevacizumab.

Her treatment course was complicated by *Mycobacterium avium-complex* (MAC) infection, elevated bilirubin and liver enzymes attributed to excessive immunoglobulin replacement therapy, and urinary tract infection (UTI) and incontinence.

Cancer genetic analysis revealed no targetable markers and primary immunodeficiency gene panel of 407 genes by Invitae was unrevealing. Lab tests revealed no evidence of Epstein-Barr Virus (EBV) infection. Post-chemotherapy imaging revealed no evidence of cancer for 1 year and 4 months, but the disease relapsed subsequently. The patient’s lung scarring requires vigilance.

**Conclusions:**

Our patient with ovarian cancer and IEI required modified treatment and prevention of complications. In cases of IEI, optimal chemotherapy should be titrated to minimize immunosuppression yet treat cancer aggressively while decreasing the risk of infection with prophylactic antibiotics and prolonged post-treatment surveillance, including pulmonary evaluation.

## Introduction

Inborn error of immunity (IEI), a category characterized by inherited disorders of the immune system, confers an increased risk for developing various cancers most commonly originating in lymphocytes, such as lymphomas (1). Increased risk of gastric cancers is also reported with an overall higher incidence of malignancies in patients with common variable immunodeficiency (CVID) compared to the general population (2). Concomitant malignancies are often the cause of death in individuals with IEI (1). Although the etiology of cancer development in IEI patients needs further exploration, it is thought to stem from impaired surveillance of the immune system, which is vital in preventing infection and oncogenesis (1). Intrinsic and extrinsic factors, many of which derive from the same molecular defects that underlie immunodeficiency itself, may increase susceptibility to cancers (3). Specifically, impaired genetic stability caused by defective DNA repair and impaired clearance of oncogenic viruses like EBV have been suggested to play a role in oncogenesis in those with IEI (4).

The management of IEIs in the setting of ovarian cancer and other comorbidities is poorly described in the literature. Ovarian cancers are the deadliest gynecological cancers in the United States and worldwide. High grade serous carcinoma is especially lethal as it recurs incurably within the remaining lifespan in 75% of patients who respond well to initial treatment (5, 6). While the knowledge of different cancers in the IEI population increases, screening modifications and treatment adjustment guidelines are not yet available. Lack of empirical evidence and guidelines discourages oncologists to offer personalized treatment plans to IEI patients largely due to the risks of infectious complications. Offering these patients a less aggressive plan of cancer chemotherapy to prevent infections may undermine the success of treatment. Here we present a patient with IEI and stage III serous ovarian cancer as well as infectious and non-infectious complications or comorbidities. The patient was treated with a dose-adjusted combination of paclitaxel/carboplatin/bevacizumab chemotherapy together with intravenous immunoglobulin (IVIG) and antibiotics.

## Case Presentation

A 60-year-old female with SADNI and ID, BRCA1/2 mutation negative, was diagnosed with stage III high grade serous ovarian carcinoma. Her diagnosis of IEI was made about 30 years before her cancer diagnosis. The patient had total serum Ig levels within normal range and normal antibody titers for diphtheria and tetanus both pre- and post-immunization. However, her pneumococcal antibody titers demonstrated an inadequate increase post-immunization. The patient has been having frequent recurrent sinopulmonary infections and pneumonias due to her immune deficiency.

Additional medical history includes asthma, hypertension, mixed connective tissue diseases, autoimmune thyroiditis, and celiac disease. Her celiac disease was confirmed by abnormal positive tissue transglutaminase IgA test with normal overall IgA titer while being treated with IVIG. Despite the diagnosis, the patient has not been following a restricted gluten-free diet. Other antibodies screening tests repeatedly showed abnormal positive results for anti-nuclear antibodies (ANA) and anti-thyroglobulin antibodies with ANA titer of 1:640 and 1:2560 at the age of 56 and 55, respectively. Eighteen months prior to her cancer diagnosis, her body mass index was normal (19.1 kg/m^2^, weight 58.2 kg), whereas it was low (17.1 kg/m^2^, weight 52 kg) two months after her diagnosis before beginning chemotherapy and it dropped more during chemotherapy (16.5 kg/m^2^, 50 kg) and has changed little since then. The patient timeline of cancer treatment, complications, and adjustments are shown in **Supplementary Table 1**. Initial immunologic profile for B cell and T cell subsets are shown in **Supplementary Table 2**. and immunoglobulin levels are listed in **Supplementary Table 3**. The patient underwent genetic testing by Invitae with a multi-cancer 84-gene panel, including BRCA1/2, which can detect most known cancer-causing variants (mutations). However, no pathogenic variants were found.

Following her diagnosis with ovarian cancer, the patient underwent robot-assisted laparoscopic total hysterectomy, bilateral salpingo-oophorectomy, resection of rectosigmoid colon and omentectomy. Prior to cancer diagnosis, her IEI treatment entailed IVIG (40 g) every two weeks (1.54 g/kg/4 weeks). Just before starting chemotherapy, her IVIG dose was increased to 80 g every 3 weeks (2.05 g/kg/4 weeks, weight 52 kg), administered 1 week prior to the first cycle of chemotherapy, to retain immunocompetence. The patient was prescribed 6 cycles of chemotherapy in total, and the doses of the chemotherapy agents were lowered to reduce the risk of IEI-related complications. The treatment included carboplatin (area under the curve [AUC] 4 instead of standard 5), paclitaxel (150 mg/m^2^ instead of standard 175 mg/m^2^) and bevacizumab (15 mg/kg) (see **Supplementary Table 1**). Additionally, the patient received colony-stimulating factor, pegfilgrastim (6 mg), through all 6 cycles to prevent febrile neutropenia.

For the first cycle of chemotherapy, bevacizumab was omitted because the patient’s vaginal cuff had not healed completely after the debulking surgery. After the first round of chemotherapy, the patient developed complications including *Mycobacterium avium-complex* (MAC) infection, hyperbilirubinemia, elevated liver function tests (LFT), and exacerbated post-surgery urinary retention. Subsequent radiographic studies (abdominal ultrasound and CT with contrast), viral hepatitis work-up, and LFT revealed no liver damage, specifically, no hepatomegaly or hepatitis. The transitory increase in LFT and hyperbilirubinemia were attributed to increased IVIG dose in conjunction with chemotherapy. Treatment for MAC infection was held due to mild symptoms and ongoing chemotherapy. To protect the liver, the patient’s IVIG dose was reduced to 60 g (1.6 g/kg/4 weeks) every 3 weeks administered 1 week before each chemotherapy cycle and continued during maintenance therapy. The doses for chemotherapy agents were not changed.

During cycle 2 and cycle 3, the patient developed a urinary tract infection (UTI) due to *Klebsiella pneumoniae*, which was treated with ceftriaxone and ciprofloxacin. This complication caused a delay of cycle 4 of her chemotherapy. Throughout cycles 4 to 6, the recurrent UTI was prevented with prophylactic trimethoprim/sulfamethoxazole and azithromycin. She also received mirabegron for symptoms of urinary incontinence. The patient experienced general chemotherapy side effects including fatigue, neuropathy of hands and feet, headache (relieved with ibuprofen), shortness of breath upon exertion, palpitation with anxiety, and abdominal pain with bowel movement. In particular, she felt nausea and severe bone pain which were managed with antiemetics and hydromorphone, respectively. Additionally, the patient experienced cyclic gastrointestinal discomforts, specifically 3-4 days of constipation followed by 1-2 days of diarrhea, after which the cycle repeated itself.

The patient managed to complete all 6 cycles of chemotherapy. **Supplementary Table 1** shows the timetable of treatment, complications, and adjustments. Upon completion of all chemotherapy, the patient’s CT scan revealed no evidence of cancer, however, her susceptibility to infections worsened. Prior to chemotherapy, her CT scans between the ages of 56-60, demonstrated small nodules in lingula, lung scarring, atelectasis, and bronchiectasis suggesting infectious/inflammatory reactions. While the imaging was consistent with latent MAC infection, no culture was performed at that time. After the first cycle of chemotherapy, the complication with MAC infection was confirmed *via* bronchoscopy. Throughout the chemotherapy, the infection was monitored with CT scans of the thorax, which initially showed nodular increase, but more recent scans showed improvement of nodular consolidation. However, after completing the chemotherapy, a CT scan demonstrated a slight increase of nodular size. Five months after the final cycle of chemotherapy, the patient had an emergency visit due to sudden onset of severe cough and hemoptysis. The bronchoscopy demonstrated clotted, dried blood, and a CT of the thorax identified consolidation of the left upper lobe lingula with nodules. The acid-fast bacilli cultures obtained via bronchoscopy with bronchoalveolar lavage grew MAC, for which the patient received azithromycin, rifabutin, and ethambutol three times per week. However, one month later, the MAC treatment was discontinued due to the feeling of exhaustion, undulant fever and myalgias, but little or no pulmonary symptoms were experienced at that time. Nine months after discontinuing therapy, a repeat CT scan of the thorax revealed further mild increase in nodular size. Another indication of increased infectious susceptibility is a high frequency of recurrent herpetic labial flares, which the patient did not have until after finishing chemotherapy. The flares are controlled with daily valacyclovir.

To mitigate recurrence of ovarian cancer, niraparib, a poly adenosine diphosphate-ribose polymerase (PARP) inhibitor, is approved for maintenance treatment of adult patients with a partial or complete response to platinum-based chemotherapy (7). Although the patient was eligible for niraparib treatment, it was not prescribed as maintenance therapy because it could exacerbate the patient’s susceptibility to infections, particularly pneumonitis, especially since the patient had neutropenia and lymphopenia (8). Before determining maintenance therapy, the patient underwent a genetic evaluation for chromosomal and somatic mutations in immune genes, which showed no pathogenic variants. The test result for EBV viral titers was also negative. Bevacizumab (15 mg/kg every 2-4 weeks), a vascular endothelial growth factor inhibitor, was prescribed for one year as maintenance therapy. However, the patient completed only 6 months of bevacizumab treatment (12 cycles) due to the MAC infection complicated by an acute episode of hemoptysis. Since completing cancer treatment, the patient was in a complete remission for 1 year and 4 months. Subsequently, the patient relapsed and progressed to stage IV high grade serous ovarian carcinoma as 3 metastatic nodules were found in her pelvis and lung. However, one nodule has recently resolved and malignant cells were absent in a recent bronchioalveolar lavage as she is currently undergoing chemotherapy, which has been paused at cycle 4 because of treatment for recurrent infections.

## Discussion

Patients with IEI who develop ovarian cancer constitute an especially rare population under-represented in the literature. The United States Immunodeficiency Network (USIDNET) reported 5 cases between the years 2011-2020 and the National Cancer Institute (NCI) reported 1 case (unknown time period). Neither registries published information describing treatment adjustments or the outcomes. Lack of clear stratification of IEI patients and personalized treatment guidelines challenges oncologists and immunologists to develop optimal treatments for patients with this rare comorbidity. The vulnerable immunity of these patients can disrupt treatments due to the risk of developing new health complications and, thereby, delay chemotherapy and reduce treatment effectiveness.

This case report provides an example of a modified treatment plan that reduced chemotherapy dosage, increased IVIG dosage and adjusted the timing of IVIG to every 3 weeks administered 1 week prior to chemotherapy. This treatment plan was formulated by a multidisciplinary team to treat ovarian cancer in the context of IEI, other comorbidities and complications that arose following the original treatment protocol.

While the literature on patients with IEI and ovarian cancers is sparse, many studies have reported on human immunodeficiency virus (HIV) and acquired immunodeficiency syndrome (AIDS) patients with non-AIDS defining cancers (NADCs) as reviewed by Ceccarelli et al. (9) These patients constitute the closest subpopulation for a relevant comparison to our patient. NADCs include cancers of the lungs, liver, breast, colon, anus, prostate, as well as Hodgkin’s lymphoma, among others. Highly active combination antiretroviral therapy (cART) offers HIV patients significant improvement in the disease morbidity, mortality, and life expectancy. However, in the settings of NADCs, a combination of cART with chemotherapy raises concerns due to the possibility of overlapping toxicities, deterioration of immune status due to chemotherapy, and potential drug-drug interactions (DDI). When overlapping toxicities are a concern, changing retroviral agent(s) or chemotherapy drug(s) is preferred to stopping cART or decreasing chemotherapy doses (10). In our case, the dose of IVIG therapy was increased (to 80 mg/3 weeks) relative to the dose prior to cancer treatment (40 mg/3 weeks) although it was lowered during chemotherapy (to 60 mg/3 weeks) due to liver toxicity and the dose of chemotherapy agents was decreased compared to the standard protocol to limit immunosuppression in a patient with IEI.

Another relevant lesson from NADC treatment is DDI between cART and chemotherapy agents. Highly active combination ART may alter pharmacology of anticancer drugs by altering metabolizing enzymes, potentially leading to decreased efficacy or increased toxicity of the co-administered chemotherapy agents (11). One of the approaches to address DDI is to use anticancer drugs that do not use the same clearance routes as immunodeficiency treatment agents. In our case, IVIG treatment didn’t represent DDI concern because antibody elimination occurs mostly through intracellular catabolism by lysosomal degradation except for pathologic conditions (12). However, DDI should be a concern when chemotherapy complications are treated pharmacologically. In our case, the patient had a MAC complication, which was treated after completion of chemotherapy to avoid potential interactions. DDI should be taken into consideration when such treatments are co-administered. For NADCs, collaborative multidisciplinary efforts between oncologists, HIV specialists, and clinical pharmacists are emphasized (13), which resembles the multidisciplinary strategy we employed in our case.

Development of clinical MAC infection after the first cycle and lung scarring after the full treatment course may necessitate in-depth pulmonary screening for infections (CT scan and then bronchoscopy if indicated) and evaluation before intervention, which was a limitation of our case. The timing of the infection suggests that chemotherapy unmasked the dormant MAC bacteria or enabled re-infection. Since MAC is ubiquitous in the environment, endogenous reactivation is difficult to confirm (14, 15). However, experimental reactivation of latent pulmonary MAC infection has been demonstrated in a mouse model (16). This observation signifies the importance of screenings for opportunistic infections in patients with immunodeficiency and past history of infection. Multiple infections, often UTIs, throughout chemotherapy is reflective of the high susceptibility incurred by immune compromised individuals, which requires close surveillance by treating physicians.

Elevated LFTs, hyperbilirubinemia, and subsequent biopsy consistent with drug-induced hepatitis were attributed to the increased dose of IVIG that may cause transaminitis (17); consequently, the subsequent dose of IVIG was reduced. This observation emphasizes the importance of a multidisciplinary team approach for such a unique presentation, which was a strength of our case.

In cases of cancer with IEI generally, the standard treatment protocol for immunocompetent individuals is recommended except a shorter regimen is preferable and a close watch should be kept for likely infections (18–20). Increasing research shows promise for combining conventional oncology drugs with off label use of repurposed drugs with known anti-tumor activity, including against chemotherapy-resistant cancer stem cells (6). This strategy could particularly benefit patients with IEI and cancer by reducing the chemotherapeutic dose while providing some antimicrobial prophylaxis. Many of these repurposed drugs are antimicrobial agents including salinomycin, monensin, ritonavir, itraconazole, nitazoxanide and ivermectin (6, 21, 22). For instance, ivermectin synergized with cisplatin to completely inhibit ovarian tumor growth of xenografts in mice compared to 1/3^rd^ inhibition with cisplatin alone (23). All treatments were well tolerated whether given singly or in combination. Growth arrest and apoptosis occurred in the tumor cells by suppressing Akt/mTOR signaling.

Currently, no evidence shows malignancies that develop in patients with IEI are particularly resistant to chemotherapy. Rather, IEI patients are prone to developing concurrent infections and are more vulnerable to developing widespread cancer, which demands aggressive therapy (19). Our case study demonstrates a multidisciplinary team involving an oncologist and immunologist that pursued aggressive chemotherapy while managing likely complications associated with IEI. The 5-year survival for general patients with epithelial ovarian cancer is about 48%, with regional high-grade serous ovarian carcinoma comprising 67.7% of cases (24). Similar case reports and multi-center studies investigating how immunodeficiency can affect treatment and prognosis of ovarian cancer are needed. Additionally, because susceptibility to infections is a side effect of chemotherapy in all patients, immunologic evaluation should be considered in all patients prior to chemotherapy regardless of whether the patient has IEI.

This case exemplifies the challenge and ongoing debate on how to treat aggressively and concurrently advanced malignancy and recurrent infections in a patient with IEI. Such as screening for dormant pulmonary infections, preventing opportunistic infections, and cooperating closely with a multidisciplinary team of physicians to allow for immediate adjustments in case of unpredictable complications. Additionally, our case demonstrates that aggressive cancer treatment should be available to the patient with ovarian cancer and IEI, as our patient managed to complete full chemotherapy and showed a good treatment response with cancer remission lasting for 1 year and 4 months. As we previously noted, high grade serous carcinoma recurs in 75% of patients who respond well to initial treatment, which unfortunately happened to our patient. Although premature cessation of maintenance therapy might increase the risk of cancer recurrence, bevacizumab was discontinued within 6 months for concern it was exacerbating hemoptysis secondary to MAC infection. The hemoptysis was likely a consequence of discontinuing MAC treatment, which the patient could not tolerate beyond one month.

Finally, the case demonstrates that IEI patients may require longer post-treatment surveillance to control their susceptibility for infectious complications (e.g., MAC, herpes).

## Patient Perspective

First, the patient emphasized the stress and anxiety after seeing the first three oncologists who were reluctant to treat her. After reviewing the literature, these oncologists were hesitant to treat her due to possible complications as they were unsure how to modify her cancer treatment to accommodate her immune deficiency and dysregulation, and yet treat her cancer effectively. Such hesitation emphasizes the need for alternative protocols for ovarian cancer treatment in patients highly susceptible to infections as the one presented here. During the patient’s initial treatment evaluation, there was no data on co-administration of IVIG and chemotherapeutic drugs for ovarian cancer. As described in the case presentation, the first cycle necessitated adjustment of the IVIG dose due to worsening liver disease.

The patient also underlined her appreciation for the close communication and cooperation among the oncologist, immunologist and infectious disease specialist who treated her during chemotherapy for ovarian cancer. The multidisciplinary collaboration for her treatment made it possible for the patient to successfully complete 6 cycles of chemotherapy. Additionally, prophylactic adjustments, such as starting pegfilgrastim, was necessary. This approach reduced the level of stress and anxiety of the patient throughout her chemotherapy.

Finally, the patient expressed the importance of disease education, nutritional consultation, and counseling for mental health while undergoing cancer treatment.

## Data Availability Statement

The original contributions presented in the study are included in the article **Supplementary Material**. Further inquiries can be directed to the corresponding author.

## Ethics Statement

Written informed consent was obtained from the participant(s) for the publication of this case report.

## Author Contributions

JM - manuscript write up, data collection, patient interview. AR - manuscript write up, literature review. RC - manuscript write up. ME - clinical research coordination. TG - pharmacy in gynecology clinic. CD, KC, BU, and SG - immunologic evaluation. AC - control of infectious diseases. CSo - control of infectious diseases. CSr - control of pulmonary infections. JD - manuscript write up, immunologic evaluation, literature review. TH - early treating immunologist, primary diagnosis with IEI. RI-K - treating oncologist. JW - currently treating immunologist, primary investigator. All authors contributed to the article and approved the submitted version.

## Conflict of Interest

The authors declare that the research was conducted in the absence of any commercial or financial relationships that could be construed as a potential conflict of interest.

## Publisher’s Note

All claims expressed in this article are solely those of the authors and do not necessarily represent those of their affiliated organizations, or those of the publisher, the editors and the reviewers. Any product that may be evaluated in this article, or claim that may be made by its manufacturer, is not guaranteed or endorsed by the publisher.
