# Corrigendum: Case report: Initial treatment adjustments and complications in ovarian cancer patient with inborn error of immunity

**DOI:** 10.3389/fonc.2022.1007616

**Published:** 2022-09-22

**Authors:** Jamila Mammadova, Anna Redden, Rachel Cruz, Boglarka Ujhazi, Sumai Gordon, Maryssa Ellison, Tyra Gatewood, Carla Duff, Anthony Cannella, Charurut Somboonwit, Chakrapol Sriaroon, Krisztian Csomos, Joseph F. Dasso, Terry Harville, Roohi Ismail-Khan, Jolan E. Walter

**Affiliations:** ^1^ Morsani College of Medicine, University of South Florida, Tampa, FL, United States; ^2^ Charles E. Schmidt College of Medicine, Florida Atlantic University, Boca Raton, FL, United States; ^3^ Division of Allergy and Immunology, Department of Pediatrics, Morsani College of Medicine, University of South Florida, Tampa, FL, United States; ^4^ Department of Pharmacy at Gynecologic and Neuro Oncology Clinics, H. Lee Moffitt Cancer Center and Research Institute, Tampa, FL, United States; ^5^ Division of Infectious Disease and International Medicine, Department of Internal Medicine, Morsani College of Medicine, University of South Florida, Tampa, FL, United States; ^6^ Division of Hematology, Department of Pathology and Laboratory Services, and Department of Internal Medicine, University of Arkansas for Medical Sciences, Little Rock, AR, United States; ^7^ Department of Cardio-Oncology, H. Lee Moffitt Cancer Center and Research Institute, Tampa, FL, United States

**Keywords:** antibody deficiency, primary immunodeficiency, ovarian cancer, case report, inborn error of immunity

In the published article, there was an error in the author list, and authors Boglarka Ujhazi, Sumai Gordon, and Krisztian Csomos were erroneously excluded. In addition, their author contributions statement has been updated. The corrected statement reads as follows:

JM - manuscript write up, data collection, patient interview. AR - manuscript write up, literature review. RC - manuscript write up. ME - clinical research coordination. TG - pharmacy in gynecology clinic. CD, KC, BU, and SG - immunologic evaluation. AC - control of infectious diseases. CSo - control of infectious diseases. CSr - control of pulmonary infections. JD - manuscript write up, immunologic evaluation, literature review. TH - early treating immunologist, primary diagnosis with IEI. RI-K - treating oncologist. JW - currently treating immunologist, primary investigator. All authors contributed to the article and approved the submitted version.

The authors apologize for these errors and state that this does not change the scientific conclusions of the article in any way. The original article has been updated.

## Publisher’s note

All claims expressed in this article are solely those of the authors and do not necessarily represent those of their affiliated organizations, or those of the publisher, the editors and the reviewers. Any product that may be evaluated in this article, or claim that may be made by its manufacturer, is not guaranteed or endorsed by the publisher.

