# Supplementary material for: Case Report: Initial Treatment Adjustments and Complications in Ovarian Cancer Patient With Inborn Error of Immunity
Source: Front Oncol. 2022 Jun 29;12:843741. doi: 10.3389/fonc.2022.843741 (PMC9278814; doi:10.3389/fonc.2022.843741)
Supplement: Supplementary file 1 [file Table_1.pdf]

SUPPLEMENTARY MATERIAL

Table 1. The timeline of cancer treatment, complications, and adjustments to treatment*

Timeline/ Interven-tion(s)	IVIG Therapy	Chemotherapy	Complication(s)	Adjustment(s)
Debulking surgery	40 g of IVIG every 2 weeks (1.54 g/kg/4 weeks, weight 52 kg)			
Chemo cycle 1, treatment day 1	80 g of IVIG every 3 weeks (2.05 g/kg/4 weeks, weight 52 kg), administered 1 week prior to chemo cycle 1	Carboplatin (area under the curve [AUC] 4), paclitaxel (150 mg/m^2), pegfilgrastim. Held bevacizumab due to incompletely healed vaginal cuff.	MAC infection, Elevated LFT and hyperbilirubinemia (thought to be secondary to IVIG in conjunction with chemotherapy), Urinary retention	Reduced IVIG from 80 mg to 60 mg per 3 weeks. Cycle 2 was postponed 1 week due to complications with MAC infection, transaminitis, and exacerbated urinary retention. No chemotherapy adjustment.
Chemo cycle 2, treatment day 51	60 g of IVIG every 3 weeks (1.6 g/kg/4 weeks, weight 50 kg), 1 week prior to cycle 2	Carboplatin (AUC 4), paclitaxel (150 mg/m^2), bevacizumab (15 mg/kg), pegfligrastim	Urinary tract infection (UTI), *Klebsiella pneumoniae*	No adjustments
Chemo cycle 3, treatment day 72	60 g of IVIG every 3 weeks (1.6 g/kg/4 weeks, weight 50 kg), 1 week prior to cycle 3	Carboplatin (AUC 4), paclitaxel (150 mg/m^2) bevacizumab (15 mg/kg), pegfligrastim	UTI infection, *Klebsiella pneumoniae*	Ceftriaxone (1-dose, 2 gm IVIP) Ciprofloxacin (5-day course, 500 mg daily)
Chemo cycle 4, treatment day 107	60 g of IVIG every 3 weeks (1.6 g/kg/4 weeks, weight 50 kg), 1 week prior to cycle 4	Carboplatin (AUC 4), paclitaxel 150 mg/m^2), bevacizumab (15 mg/kg), pegfligrastim	Urinary incontinence	Prophylactic trimethoprim/sulfamethox-azole (dosage unknown) and azithromycin (continuous, 250 mg daily). Mirabegron (continuous, 25 mg daily) for urinary incontinence.
Chemo cycle 5, treatment day 128	60 g of IVIG every 3 weeks (1.6 g/kg/4 weeks, weight 50 kg), 1 week prior to cycle 5	Carboplatin (AUC 4), paclitaxel (150 mg/m^2), bevacizumab (15 mg/kg), pegfligrastim	General side effects including fatigue, neuropathy of hands/ feet, headache (treated with ibuprofen and famotidine), dyspnea with exertion, anxiety, abdominal pain with bowel movement, bone pain, and cyclic GI discomfort.	Prophylactic trimethoprim/sulfamethoxazo le (dosage unknown) and azithromycin (continuous, 250 mg daily). Famotidine-ibuprofen (PRN: pain, 800 mg – 26.6 mg, TID) for neuropathy relief. Hydromorphone (3-day course, 2 mg daily) for bone pain relief.
Chemo cycle 6, treatment day 149	60 g of IVIG every 3 weeks (1.6 g/kg/4 weeks, weight 50 kg), 1 week prior to cycle 6	Carboplatin (AUC 4), paclitaxel (150 mg/m^2), bevacizumab (15 mg/kg), pegfligrastim	Same as in cycle 5	Same as in cycle 5
Post-treatment maintenance	60 g of IVIG every 3 weeks (1.6 g/kg/4	Bevacizumab (15 mg/kg, every 2-4 weeks), prescribed for 1 year as	MAC infection Herpes labialis flares	One dose of azithromycin (500 mg), ethambutol (1200 mg) and rifabutin (300 mg)

	weeks, weight 50 kg) every 3 weeks	maintenance therapy but discontinued after 6 moths (completed 12 cycles) due to MAC infection complicated by hemoptysis.		every Monday, Wednesday and Friday for 6 months for MAC infection but discontinued after 1 month due to poor tolerance and no pulmonary complaints. Valaciclovir (continuous, 500 mg daily) for prevention of herpes flares.

*Chemotherapy drugs were given once per cycle via port, bevacizumab and ceftriaxone were given via intravenous piggybank setup (IVIP), and all of the remaining medications were given orally unless otherwise indicated.

(**Supplementary Tables 2 and 3** are shown below)

Table 2. Patient's immunologic profile, B and T cell subsets prior to treatment (age 61 years)

B cell subsets

B cell subsets			Frequency of total B cells (%)	Frequency: averages of HDs and HD Reference range	Absolute count (Count/ul)	Count: averages of HDs and HD Reference range
CD19+ B cells			8.08	10.51<br>(5.61-16.0)	113	191<br>(89-414)
PC (CD38+, CD27+)			0.68	0.81<br>(0.08-3.9)	1	1<br>(1-7)
Naïve (CD27-, IgD+)	Total Naïve B		83.43	67.22<br>(40.7-83.4)	94	125<br>(36-313)
		AN	4.69	5.05<br>(1.5-11.1)	5	16<br>(1-49)
		MN	74.58	60.7<br>(36.8-76.3)	79	142<br>(15-379)
		Transitional	3.1	2.94<br>(1-6.4)	3	8<br>(1-25)
Memory (CD27+, IgD-/+)	Total Memory B		10.12 (L)	28.08<br>(14.8-51.2)	11 (L)	51<br>(17-165)
		NSM	1.16 (L)	2.7<br>(6.0-25.1)	1	3<br>(1-10)
		CSM	5.29	12.1<br>(3.6-19.7)	1	7<br>(1-24)
		MZ	2.57 (L)	44.9<br>(21.3-72.7)	1 (L)	22.54<br>(3-30)
DN (CD27-, IgD-)			5.35	4.81<br>(1.7-16.2)	6	8<br>2-34
CD21low (CD19+ CD21-)			2.32	4.85<br>(1.67-9.8)	3	9<br>3-33

(L) = Low, (HD) = Healthy Donor

Plasmablasts/plasma cells (PC): $CD45^{+}$ $CD19^{+}$ $CD38^{high}$ $CD27^{high}$

Memory B cells: $CD45^{+}$ $CD19^{+}$ $CD27^{+}$ $IgD^{-/+}$

- Switched memory (CSM): $CD45^{+}$ $CD19^{+}$ $CD27^{+}$ IgM^{-} IgD^{-}
- Non-switched memory. (NSM): $CD45^{+}$ $CD19^{+}$ $CD27^{+}$ IgM^{+} IgD^{-}
- Marginal Zone-like (MZ): $CD45^{+}$ $CD19^{+}$ $CD27^{+}$ IgM^{+} IgD^{+}

Naive B cells: $CD45^{+}$ $CD19^{+}$ $CD27^{-}$ IgD^{+}

- Transitional (Trans): $CD45^{+}$ $CD19^{+}$ $CD27^{-}$ IgD^{+} $CD24^{high}$ $CD38^{high}$
- Mature naive (MN): $CD45^{+}$ $CD19^{+}$ $CD27^{-}$ IgD^{+} $CD24^{mid}$ $CD38^{mid}$
- $CD38^{low}$ naïve (AN): $CD45^{+}$ $CD19^{+}$ $CD27^{-}$ IgD^{+} $CD38^{low}$

Double negative B cells (DN): $CD45^{+}$ $CD19^{+}$ $CD27^{-}$ IgD

CD21low B cells: $CD45^{+}$ $CD21^{low}$ $CD19^{high}$

T cell subsets

T cell subsets			Frequency of total B cells (%)	Frequency: averages of HDs and HD Reference range	Absolute count (Count/ul)	Count: averages of HDs and HD Reference range
CD3 T cells			76.7	72.24	113 (L)	1251
				52-83		740-2400
CD4 T cells	Total CD4		63.2	57.9	676	728
				(42.9-75)		(256-1459)
		CD4 RTE	23.1	38.8	156	261
				(21.1-52)		(84-622)
		CD4 Naïve	38	50.2	257	360
				(27.4-70)		(114-828)
		CD4 CM	54.8	43.3	370	320
				(26-69.3)		(59-664)
		CD4 EM	6.94	5.6	47	42
				(2-9)		(4-76)
CD8 T cells	Total CD8		34	34.9	363	449
				(21.3-50)		(144-1375)
		CD8 Naïve	35.3	47	128	221
				(29.3-69)		(49-843)
		CD8 CM	63.9	47.4	232	204
				(21.1-66.6)		(59-541)
		CD8 EM	0.79 (L)	4.1	3	20
				(2-16)		(2-76)
Treg			5.29	6.6	37	53
				(4.1-8.2)		(10-100)
Tfh			11.83	10.1	83	72
				(6-16)		(23-120)

(L) = Low, (HD) = Healthy Donor

T cells: CD45+ CD3+

CD4 T cells: CD45+ CD3+ CD8− CD4+

Recent thymic emigrant (RTE): CD45+ CD3+ CD8− CD4+ CD31+ CD45RA−

Naive T cells: CD45+ CD3+CD8− CD4+ CCR7+ CD45RA+

Central memory T cells (CM): CD45+ CD3+CD8− CD4+ CCR7+ CD45RA−

Effector memory T cells (EM): CD45+ CD3+CD8− CD4+ CCR7− CD45RA−

CD8 T cells: CD45+ CD3+ CD8+ CD4−

Naive T cells: CD45+ CD3+ CD8+ CD4− CCR7+ CD45RA+

Central memory T cells (CM): CD45+ CD3+ CD8+ CD4− CCR7+ CD45RA−

Effector memory T cells (EM): CD45+ CD3+ CD8+ CD4− CCR7− CD45RA−

Regulatory T cells (Treg): CD45+ CD4+ CD25high CD127low

Follicular helper T cells (Tfh): CD45+ CD4+ CD25low/mid CD127mid/+ CXCR5+ CD45RA−

Table 3. Patient's immunoglobulins levels prior to chemotherapy while on IVIG (age 59 years)

Lab Test	Result	Range (mg/dL)
Serum IgA	252	44 - 441
Serum IgG	2,000	528 – 2,190
Serum IgM	132	48 – 226

Note: This lab result was obtained while the patient was on IVIG replacement therapy.
